# Supplementary material for: BDE-47, -99, -209 and Their Ternary Mixture Disrupt Glucose and Lipid Metabolism of Hepg2 Cells at Dietary Relevant Concentrations: Mechanistic Insight through Integrated Transcriptomics and Proteomics Analysis
Source: Int J Mol Sci. 2022 Nov 21;23(22):14465. doi: 10.3390/ijms232214465 (PMC9697228; doi:10.3390/ijms232214465)
Supplement: Supplementary file 1 [file ijms-23-14465-s001.zip › Supplementary Figures.pdf]

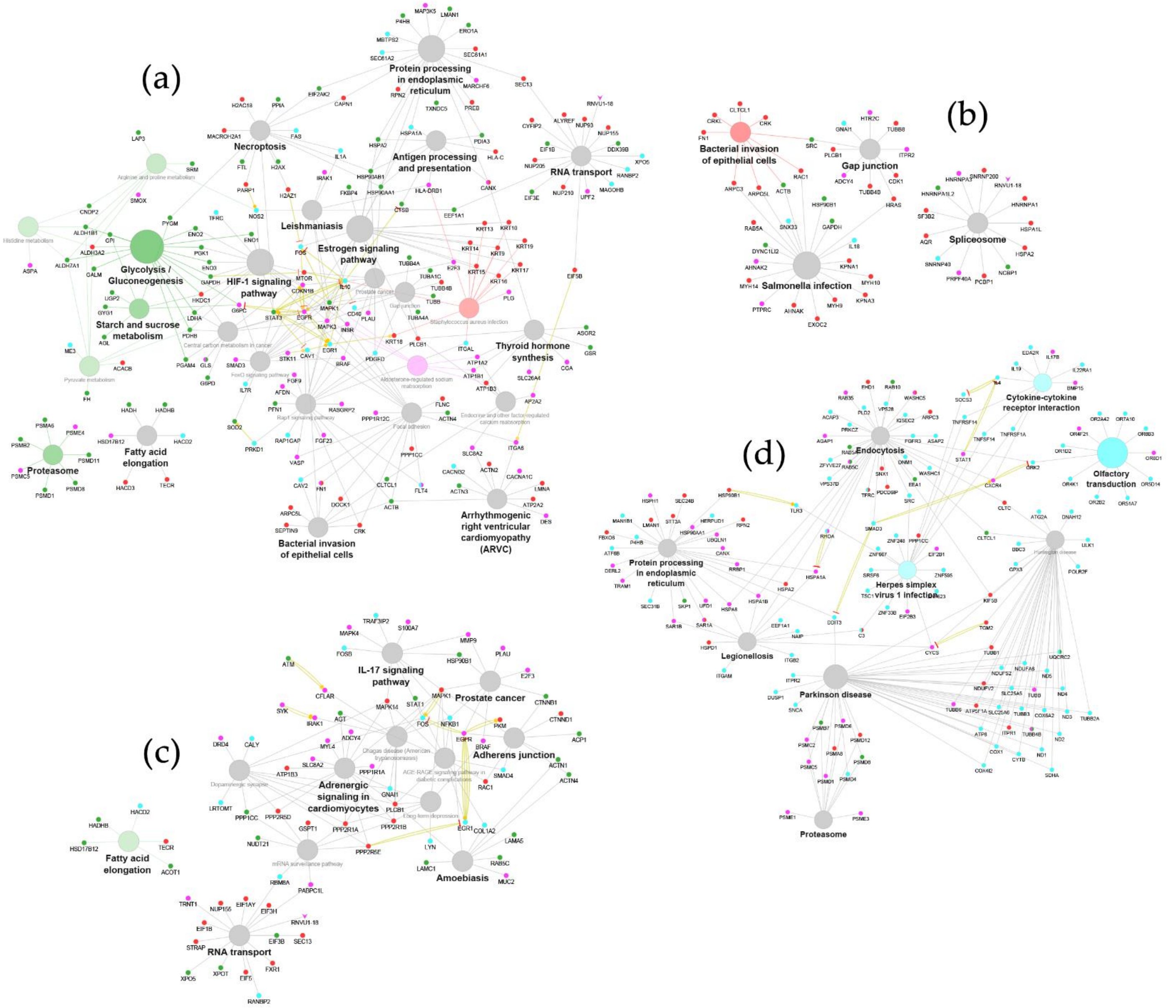

Figure S1 – Enriched KEGG pathways in HepG2 cells treated for 72h with (a) BDE-47, (b) BDE-99, (c) BDE-209 or (d) MIX at 1 nM. DEGs and DEPs featured in enriched terms are shown and colored as follows: magenta, up-regulated DEGs; cyan, down-regulated DEGs; red, up-regulated DEPs; green, down-regulated DEPs. Enriched terms are colored accordingly to the prevalence of one of the four groups. The size of the enriched terms is proportional to the significance.

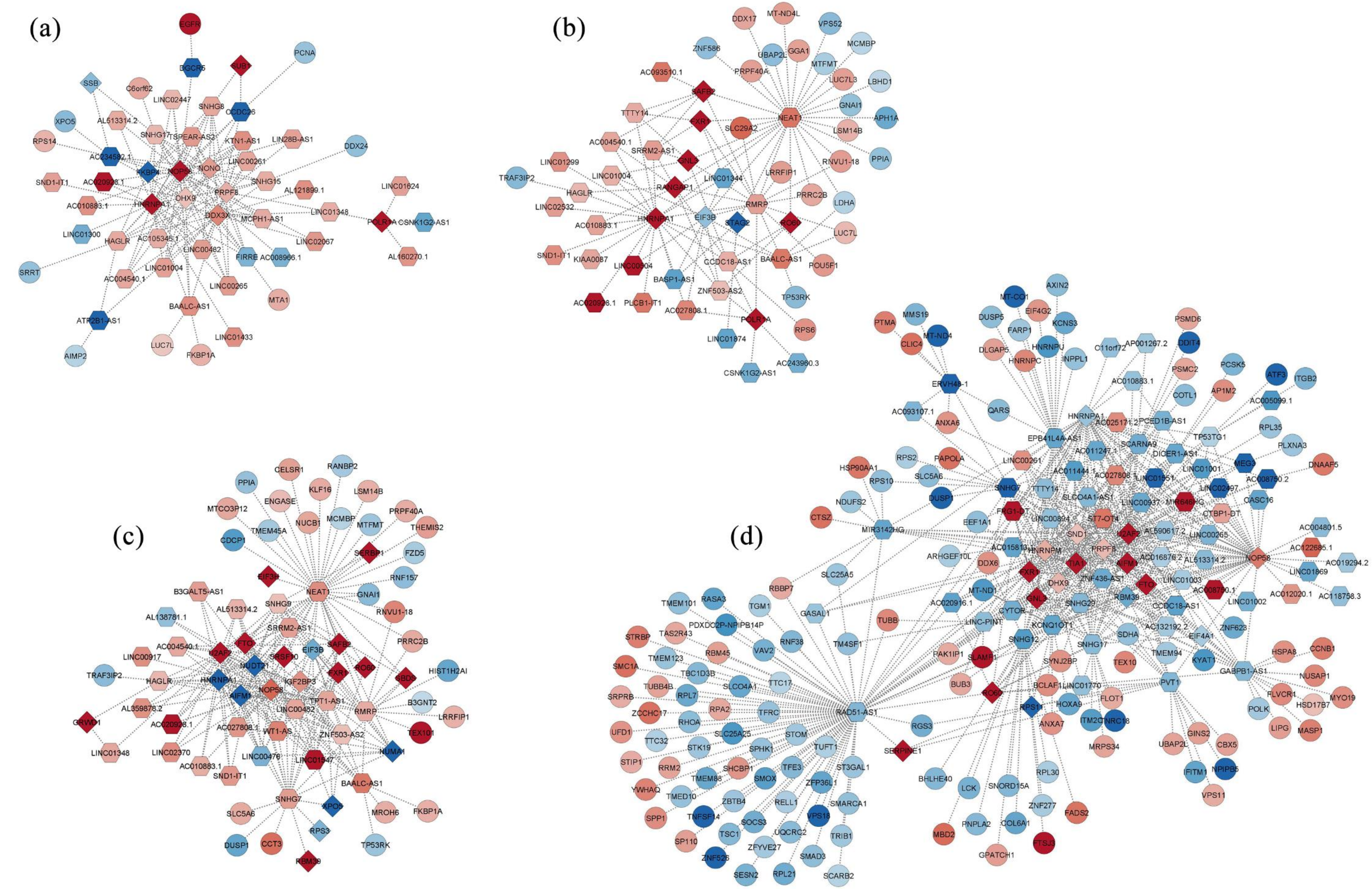

Figure S2 – Interaction networks between deregulated DELs (hexagons), DEGs (circles) and DEPs (diamonds), as inferred by publicly available validated experiments, in HepG2 cells treated for 72h with (a) BDE-47, (b) BDE-99, (c) BDE-209 or (d) MIX at 1 nM. Shades of blue and red represent, respectively, the magnitude of down- or up-regulation.
