# Supplementary material for: BDE-47, -99, -209 and Their Ternary Mixture Disrupt Glucose and Lipid Metabolism of Hepg2 Cells at Dietary Relevant Concentrations: Mechanistic Insight through Integrated Transcriptomics and Proteomics Analysis
Source: Int J Mol Sci. 2022 Nov 21;23(22):14465. doi: 10.3390/ijms232214465 (PMC9697228; doi:10.3390/ijms232214465)
Supplement: Supplementary file 1 [file ijms-23-14465-s001.zip › Supplementary Tables.pdf]

Table S1 – List of the differentially expressed lncRNAs identified in HepG2 cells following treatment for 72h with BDE-47, -99, -209 and their ternary mixture (MIX) at 1 nM. Values are log_2FC ($p<0.01$ and FDR < 5%).

Gene name	Entrez Gene ID	BDE-47	BDE-99	BDE-209	MIX
**AC004080.1**					-0.957
**AC004540.1**		0.5156	0.5266	0.5204	
**AC004801.5**					-0.6
**AC005523.2**		0.6709	0.7826	0.8516	
**AC008394.1**			0.8986		
**AC008750.1**					1.436
**AC008750.2**					-1.335
**AC008966.1**		-0.6868	0.5765		
**AC010883.1**		0.6725	0.5443	0.4861	-0.532
**AC011247.1**					-0.787
**AC011444.1**					-0.895
**AC012020.1**					0.6343
**AC015813.1**					-0.829
**AC016876.2**					-0.412
**AC019257.1**				-0.8174	
**AC019294.2**					-0.48
**AC020916.1**					-0.847
**AC020928.1**		1.2854	1.5202	1.6698	
**AC023355.2**					1.0084
**AC025171.2**					0.7014
**AC027808.1**			0.7389	0.6327	0.7409
**AC040168.1**					0.738
**AC093510.1**		0.686	0.845		
**AC093817.1**					-0.982
**AC096577.1**			-0.8077		
**AC100803.2**					-0.786
**AC103739.2**					-0.502
**AC105345.1**		0.4812			
**AC118758.3**				0.06069	-0.562
**AC122685.1**					0.9657
**AC132192.2**					-0.461
**AC234582.1**		-1.0242			
**AC243960.3**			-0.725		
**AC245060.2**				0.5548	
**AC245297.4**		0.4847		0.5834	
**AL121899.1**		0.6708	0.6105		
**AL136090.1**					-0.608
**AL138781.1**				-0.5563	
**AL160270.1**		0.6497			
**AL359878.2**			0.668	0.6619	
**AL449403.2**					-0.589
**AL513314.2**		0.4974		0.4518	-0.597
**AL590617.2**					-0.505
**AP001267.2**					-0.472
**AP001999.1**					1.448

**ASAP1-IT1**	29065		1.0789		
**ATP2B1-AS1**	338758	-1.0028			
**AUXG01000058.1**			0.738		
**B3GALT5-AS1**	114041			0.5876	
**BAALC-AS1**	100499183	0.68	0.8284	0.8266	
**BASP1-AS1**	285696		-0.8496		
**BX649632.1**			0.6112		
**C11orf72**	100505621				-0.548
**CASC16**	643714				-0.807
**CCDC18-AS1**	100131564		0.4775		-0.846
**CCDC26**	728724	-1.039			
**CSNK1G2-AS1**	255193	-0.8093	-0.7341		
**CSTF3-DT**	338739	-0.8861	-0.8184		
**CTBP1-DT**	92070				0.5424
**CU634019.6**		0.4277			
**CYTOR**	112597				-0.728
**DGCR5**	26220	-1.0488			
**DICER1-AS1**	400242				-0.794
**DLGAP1-AS5**	284215	-0.6672			
**EPB41L4A-AS1**	114915				-0.811
**ERVH48-1**	90625				-1.113
**FAM135A-AS1**	105377849				1.0397
**FIRRE**	286467	-0.6693			
**FOXN3-AS2**	29018				1.1009
**FRG1-DT**	728339				1.0858
**GABPB1-AS1**	100129387				-0.621
**GASAL1**	401472				-0.507
**HAGLR**	401022	0.5126	0.4095	0.4187	
**HOXB-AS2**	100874350				-1.383
**KCNQ1OT1**	10984				-0.81
**KIAA0087**	9808		0.5323		
**KTN1-AS1**	100129075	0.6752			
**LIN28B-AS1**	100113403	0.5607			
**LINC00260**	84719				-0.971
**LINC00261**	140828	0.5172			0.6618
**LINC00265**	349114	0.6038			-0.667
**LINC00316**	388830		-0.7113		
**LINC00476**	100128782			-0.6885	
**LINC00482**	284185	0.5917		0.5153	
**LINC00597**	81698			0.5952	
**LINC00894**	100272228				-0.544
**LINC00917**	732275		0.7193	0.6749	
**LINC00937**	389634				-0.779
**LINC01001**	100133161				-0.911
**LINC01003**	100128822				-0.442
**LINC01004**	100216546	0.5705	0.4576		
**LINC01061**	401149				-0.759
**LINC01114**	284998		0.6454		
**LINC01214**	101928022	-0.6761			

**LINC01299**	286186		0.6596		
**LINC01300**	731779	-0.733			
**LINC01344**	400799		-0.8165		
**LINC01348**	731656	0.4767		0.522	
**LINC01381**	106144568			1.1779	
**LINC01433**	728228	0.6833			
**LINC01546**	100129464	-0.8232			
**LINC01547**	84536			1.1063	
**LINC01551**	387978				-1.998
**LINC01624**	401289	0.5733			
**LINC01770**	102724312				-0.552
**LINC01869**	284365				-0.787
**LINC01874**	727944		-0.821		
**LINC01881**	728323				-0.454
**LINC02067**	101243545	0.6242			
**LINC02138**	400558				-0.807
**LINC02370**	338797		0.7439	0.6921	
**LINC02371**	107984539	0.6574			
**LINC02447**	730971	0.432			
**LINC02497**	102723778				-2.256
**LINC02532**	100422737		0.594		
**LINC02825**	283435				-1.409
**LINC-PINT**	378805				-0.57
**LOC100126784**	100126784				-0.733
**LOC114224**	114224	0.4335	0.4928		
**MCF2L-AS1**	100289410				0.6328
**MCPH1-AS1**	100507530	0.5004			
**MEG3**	55384				-1.211
**MIR3142HG**	107075116				-0.805
**MIR646HG**	284757				1.3888
**NEAT1**	283131		0.8549	0.6954	
**OOEP-AS1**	106478938		0.8068		
**PCED1B-AS1**	100233209				-0.695
**PLCB1-IT1**	100874337		0.839		
**PVT1**	5820				-0.633
**RAD51-AS1**	100505648				-0.641
**RMRP**	6023		0.5664	0.4904	
**SCARNA9**	619383				-0.735
**SLCO4A1-AS1**	100127888				-0.626
**SND1-IT1**	27099	0.5676	0.6856	0.62	
**SNHG12**	85028				-0.904
**SNHG15**	285958	0.4854			
**SNHG17**	388796	0.4177			-0.604
**SNHG29**	125144				-0.621
**SNHG32**	50854				-0.679
**SNHG7**	84973			0.586	-1.078
**SNHG8**	100093630	0.5299			
**SNHG9**	735301			0.3624	
**SRRM2-AS1**	100128788		0.5883	0.5751	

**ST7-OT4**	338069				0.843
**TP53TG1**	11257				-0.383
**TPT1-AS1**	100190939			0.5327	
**TSL**	790953				-0.884
**TSPEAR-AS2**	114043	0.607			
**TTLL1-AS1**	100506679				-0.676
**TTTY13**	83868				-0.932
**TTTY14**	83869		0.4959		-0.579
**TTTY18**	252950	0.8297	0.686	0.7064	
**WT1-AS**	51352			0.7635	
**ZNF436-AS1**	148898				-0.75
**ZNF503-AS2**	100131213		0.3868	0.3652	

Table S2 – Common features expressed at gene and protein expression level in HepG2 cells treated for 72h with BDE-47, -99, -209 and their ternary mixture (MIX) at 1 nM.

BDE47	BDE99	BDE209	MIX
CANX, DDX18, FKBP1A, FN1, GLS, HEATR1, KRT1, PMPCA, PRPF8, RHOC, SRRT	HDGF, POTEI, PRDX1, PSMD10, PSME4	AKR1D1, CYB5R3, PRPF40A, PSMD6, SAFB2	ACLY, C3, CTNNB1, CTSA, DARS2, DHX9, FARP1, FKBP1A, GLG1, HNRNPA1, HSPG2, LAMB1, MMS19, MRPS23, MSH2, MYBBP1A, MYO1C, NARS1, NUDT5, PDHX, PGM3, POR, PTP4A1, RAB5C, RBM39, RPA2, RPL21, SAR1A, SCARB2, SDF2L1, SEPTIN9, SERPINE1, SLC2A3, TFRC, TUBB4B, UQCRC2, VAV2, WASHC5

Table S3 – Significantly enriched KEGG pathways in HepG2 treated with BDE-47, -99, -209 and their ternary mixture (MIX) at 1 nM for 72h. Proteins (regular font) and Genes (italic font) associated to each enriched KEGG pathways are listed for each experimental condition in decreasing order of significance with corresponding p-values indicated in each cell. Up-regulated entities are in red and down-regulated entities are in blue.

KEGG Pathways	BDE-47	BDE-99	BDE-209	MIX
Glycolysis/Gluconeogenesis	p-value = 5.01E-05 ALDH3A2, HKDC1, ALDH1B1, ALDH7A1, ENO1, ENO2, ENO3, GALM, GAPDH, GPI, LDHA, PDHB, PGAM4, PGK, *G6PC*			
Olfactory transduction				p-value = 1.79E-04 *OR4F21, OR8D1, GRK2, OR1D2, OR2A42, OR2B2, OR4K1, OR51A7, OR5D14, OR7A10, OR8B3*
HIF-1 signaling pathway	p-value = 6.93E-04 HKDC1, MTOR, ENO1, ENO2, ENO3, GAPDH, LDHA, MAPK1, PDHB, PGK1, STAT3, *CDKN1B, EGFR, INSR, MAPK3, NOS2, TFRC*			
Estrogen signaling pathway	p-value = 9.10E-04 KRT10, KRT13, KRT14, KRT15, KRT16, KRT17, KRT18, KRT19, KRT9, PLCB1, FKBP4, HSP90AA1, HSP90AB1, HSPA2, MAPK1, *EGFR, MAPK3, FOS, HSPA1A*			
Parkinson disease				p-value = 1.41E-03 ATP5F1A, ITPR1, KIF5B, NDUFV2, PSMA8, PSMD12, TUBB1, PSMB7, PSMD8, TUBB4B, UQCRC2, *CYCS, PSMC2, PSMC5, PSMD1, PSMD6, TUBB, TUBB4B, TUBB6, ATP6, COX1, COX4I2, COX6A2, CYTB, DDIT3, DUSP1, ITPR2, ND1, ND2, ND3, ND4, ND5, NDUFA6, NDUFS2, PSMD4, SDHA, SLC25A5, SLC25A6, SNCA, TUBB2A, TUBB3, UQCRC2*
Salmonella infection		p-value = 6.31E-03 AHNAK, ARPC3, ARPC5L, EXOC2, HRAS, KPNA1, KPNA3, MYH10, MYH14, MYH9, RAB5A, RAC1, ACTB, DYNC1LI2, GAPDH, HSP90B1, *AHNAK2, PTPRC, IL18, SNX33*		
Pyruvate metabolism	p-value = 8.17E-03 ACACB, ALDH3A2, ALDH1B1, ALDH7A1, FH, LDHA, PDHB, *ME3*			
Staphylococcus aureus infection	p-value = 9.78E-03 KRT10, KRT13, KRT14, KRT15, KRT16, KRT17, KRT18, KRT19, KRT9, *HLA-DRB1, PLG, IL10, ITGAL*			
Protein processing in endoplasmic reticulum	p-value = 3.16E-03 CANX, CAPN1, PREB, RPN2, SEC13, SEC61A1, EIF2AK2, ERO1A, HSP90AA1, HSP90AB1, HSPA2, LMAN1, P4HB, PDIA3, TXNDC5, *CANX, MAP3K5, MARCHF6,*			p-value = 2.02E-02 FBXO6, HSP90B1, HSPA2, LMAN1, RPN2, SAR1A, SEC24B, STT3A, SKP1, *CANX, DERL2, HSP90AA1, HSPA1A, HSPA1B, HSPA8, HSPH1, RRBP1, SAR1A, SAR1B, TRAM1, UBQLN1, UFD1,*

	HSPA1A, MBTPS2, SEC61A2			*ATF6B, DDIT3, HERPUD1, MAN1B1, P4HB, SEC31B*
Central carbon metabolism in cancer	p-value = 1.62E-02 HKDC1, MTOR, G6PD, GLS, LDHA, MAPK1, PDHB, PGAM4, *EGFR, GLS, MAPK3*			
Legionellosis				p-value = 1.73E-02 C3, HSPA2, HSPD1, SAR1A, *CYCS, HSPA1A, HSPA1B, HSPA8, SAR1A, SAR1B, C3, EEF1A1, ITGAM, ITGB2, NAIP*
Fatty acid elongation	p-value = 1.74E-02 HACD3, TECR, HADH, HADHB, *HSD17B12, HACD2*		p-value = 2.66E-02 TECR, ACOT1, HADHB, HSD17B12, *HACD2*	
Adherens junction			p-value = 1.83E-02 CTNND1, MAPK1, RAC1, ACP1, ACTN1, ACTN4, CTNNB1, *EGFR, SMAD4*	
Starch and sucrose metabolism	p-value = 1.87E-02 HKDC1, AGL, GPI, GYG1, PYGM, UGP2, *G6PC*			
Adrenergic signaling in cardiomyocytes			p-value = 1.88E-02 ATP1B3, MAPK1, MAPK14, PLCB1, PPP2R1A, PPP2R1B, PPP2R5D, PPP2R5E, AGT, PPP1CC, *ADCY4, MYL4, PPP1R1A, SLC8A2, GNAI1*	
Herpes simplex virus 1 infection				p-value = 1.90E-02 C3, PPP1CC, *CYCS, EIF2B1, EIF2B3, STAT1, C3, SOCS3, SRC, SRSF6, TLR3, TNFRSF14, TNFRSF1A, TNFSF14, TSC1, ZNF248, ZNF33B, ZNF595, ZNF623, ZNF667*
Proteasome	p-value = 4.27E-02 PSMA6, PSMB2, PSMD1, PSMD11, PSMD8, *PSMC5, PSME4*			p-value = 2.22E-02 PSMA8, PSMD12, PSMB7, PSMD8, *PSMC2, PSMC5, PSMD1, PSMD6, PSME1, PSME3, PSMD4*
Arginine and proline metabolism	p-value = 2.25E-02 ALDH3A2, ALDH1B1, ALDH7A1, CNDP2, LAP3, SRM, *SMOX, NOS2*			
Endocytosis				p-value = 2.48E-02 ARPC3, CLTC, EHD1, HSPA2, KIF5B, PDCD6IP, SNX1, TFRC, WASHC5, CLTCL1, EEA1, RAB10, RAB5A, RAB5C, *AGAP1, CXCR4, HSPA1A, HSPA1B, HSPA8, RAB35, RAB5C, RHOA, WASHC5, ACAP3, ASAP2, DNM1, FGFR3, GRK2, IQSEC2, PLD2, PRKCZ, SMAD3, SRC, TFRC, VPS28, VPS37D, WASHC1, ZFYVE27*
AGE-RAGE signaling pathway in diabetic complications			p-value = 2.59E-02 MAPK1, MAPK14, PLCB1, RAC1, AGT, STAT1, *COL1A2, EGR1, NFKB1, SMAD4*	
RNA transport	p-value = 4.43E-02 ALYREF, CYFIP2, EIF5B, NUP155, NUP205, NUP210, NUP93, SEC13, DDX39B, EEF1A1, EIF1B, EIF3E, *RNVU1-18, UPF2, MAGOHB, RANBP2, XPO5*		p-value = 2.59E-02 EIF1AY, EIF1B, EIF3H, EIF5, FXR1, NUP155, SEC13, STRAP, EIF3B, XPO5, XPOT, *PABPC1L, RNVU1-18, TRNT1, RANBP2, RBM8A*	
Chagas disease (American trypanosomiasis)			p-value = 2.61E-02 MAPK1, MAPK14, PLCB1, PPP2R1A, PPP2R1B,	

			CFLAR, IRAK1, FOS, GNAI1, NFKB1	
Gap junction	p-value = 4.33E-02 PLCB1, TUBB4B, MAPK1, TUBA1C, TUBA4A, TUBB, TUBB4A, EGFR, MAPK3, PDGFD	p-value = 2.77E-02 CDK1, HRAS, PLCB1, TUBB4B, TUBB8, SRC, ADCY4, HTR2C, ITPR2, GNAI1		
Dopaminergic synapse			p-value = 2.79E-02 MAPK14, PLCB1, PPP2R1A, PPP2R1B, PPP2R5D, PPP2R5E, PPP1CC, DRD4, CALY, FOS, GNAI1, LRTOMT	
IL-17 signaling pathway			p-value = 2.87E-02 MAPK1, MAPK14, HSP90B1, MAPK4, MMP9, S100A7, FOS, FOSB, NFKB1, TRAF3IP2	
Arrhythmogenic right ventricular cardiomyopathy (ARVC)	p-value = 3.05E-02 ACTN2, ATP2A2, LMNA, ACTB, ACTN3, CACNA1C, DES, ITGA6, SLC8A2, CACNG2			
Long-term depression			p-value = 3.06E-02 MAPK1, PLCB1, PPP2R1A, PPP2R1B, BRAF, GNAI1, LYN	
Histidine metabolism	p-value = 3.22E-02 ALDH3A2, ALDH1B1, ALDH7A1, CNDP2, ASPA			
mRNA surveillance pathway			p-value = 3.37E-02 GSPT1, PPP2R1A, PPP2R1B, PPP2R5D, PPP2R5E, NUDT21, PPP1CC, PABPC1L, RBM8A	
Bacterial invasion of epithelial cells	p-value = 4.11E-02 ARPC5L, CRK, DOCK1, FN1, SEPTIN9, ACTB, CLTCL1, FN1, CAV1, CAV2	p-value = 4.03E-02 ARPC3, ARPC5L, CLTCL1, CRK, CRKL, FN1, RAC1, ACTB, SRC,		
Prostate cancer	p-value = 4.40E-02 MTOR, HSP90AA1, HSP90AB1, MAPK1, BRAF, CDKN1B, E2F3, EGFR, MAPK3, PLAU, PDGFD		p-value = 4.15E-02 MAPK1, CTNNB1, HSP90B1, BRAF, E2F3, EGFR, MMP9, PLAU, NFKB1	
Thyroid hormone synthesis	p-value = 4.26E-02 ATP1B3, CANX, PLCB1, ASGR2, GSR, ATP1A2, ATP1B1, CANX, CGA, SLC26A4			
Aldosterone-regulated sodium reabsorption	p-value = 4.30E-02 ATP1B3, MAPK1, ATP1A2, ATP1B1, INSR, MAPK3			
Cytokine-cytokine receptor interaction				p-value = 4.52E-02 BMP15, CXCR4, IL17B, EDA2R, IL19, IL22RA1, IL4, TNFRSF14, TNFRSF1A, TNFSF14
Rap1 signaling pathway	p-value = 4.56E-02 CRK, PLCB1, ACTB, MAPK1, PFN1, AFDN, BRAF, EGFR, FGF23, FGF9, FLT4, INSR, MAPK3, RASGRP2, VASP, FLT4, ITGAL, PDGFD, PRKD1, RAP1GAP			
Focal adhesion	p-value = 4.62E-02 CRK, DOCK1, FLNC, FN1, PPP1CC, ACTB, ACTN4, MAPK1, BRAF, EGFR, FLT4, FN1, ITGA6, MAPK3, PPP1R12C, VASP, CAV1, CAV2, FLT4, PDGFD			

Endocrine and other factor-regulated calcium reabsorption	p-value = 4.63E-02 ATP1B3, PLCB1, CLTCL1, *AP2A2, ATP1A2, ATP1B1, SLC8A2*			
FoxO signaling pathway	p-value = 4.71E-02 MAPK1, SOD2, STAT3, *BRAF, CDKN1B, EGFR, G6PC, INSR, MAPK3, SMAD3, STK11, IL10, IL7R*			
Antigen processing and presentation	p-value = 4.71E-02 CANX, HLA-C, CTSB, HSP90AA1, HSP90AB1, HSPA2, PDIA3, *CANX, HLA-DRB1, HSPA1A*			
Leishmaniasis	p-value = 4.73E-02 EEF1A1, MAPK1, *HLA-DRB1, IRAK1, MAPK3, FOS, IL10, IL1A, NOS2*			
Necroptosis	p-value = 4.80E-02 CAPN1, H2AC18, H2AZ1, MACROH2A1, PARP1, EIF2AK2, FTL, H2AX, HSP90AA1, HSP90AB1, PPIA, PYGM, STAT3, *FAS, IL1A*			
Amoebiasis			p-value = 4.80E-02 PLCB1, ACTN1, ACTN4, LAMA5, LAMC1, RAB5C, *MUC2, COL1A2, NFKB1*	
Spliceosome		p-value = 4.84E-02 AQR, HNRNPA1, HSPA1L, HSPA2, PCBP1, SF3B2, SNRNP200, HNRNPA1L2, NCBP1, *HNRNPA3, PRPF40A, RNVU1-18, SNRNP40*		
Huntington disease				p-value = 5.00E-02 ATP5F1A, CLTC, ITPR1, KIF5B, NDUFV2, TGM2, TUBB1, CLTCL1, TUBB4B, UQCRC2, *CYCS, TUBB, TUBB4B, TUBB6, ATG2A, ATP6, BBC3, COX1, COX4I2, COX6A2, CYTB, DNAH12, GPX3, ND1, ND2, ND3, ND4, ND5, NDUFA6, NDUFS2, POLR2F, SDHA, SLC25A5, SLC25A6, TUBB2A, TUBB3, ULK1, UQCRC2*

Table S4 – Significantly enriched GSEA Hallmark gene sets in HepG2 treated with BDE-47, -99, -209 and their ternary mixture (MIX) at 1 nM for 72h. The number of entities (DEGs and DEPs), the Normalized Enrichment Score (NES) and the nominal (NOM) p-value are indicated for each term. FDR is < 25% for all terms.

Hallmarks	Category	#Entities	NES	NOM p-value
**BDE-47**				
GLYCOLYSIS	metabolic	20	-2.21	0.0020
FATTY_ACID_METABOLISM	matabolic	24	-2.15	<0.0001
KRAS_SIGNALING_DN	signaling	9	1.97	0.0081
MTORC1_SIGNALING	signaling	25	-1.93	0.010
REACTIVE_OXYGEN_SPECIES_PATHWAY	pathway	8	-1.92	0.0081
UV_RESPONSE_UP	DNA damage	18	-1.90	0.017
HYPOXIA	pathway	22	-1.85	0.012
ALLOGRAFT_REJECTION	immune	12	-1.62	0.049
**BDE-99**				
EPITHELIAL_MESENCHYMAL_TRANSITION	development	8	2.00	0.0041
MTORC1_SIGNALING	signaling	14	-1.90	0.0059
PI3K_AKT_MTOR_SIGNALING	signaling	11	1.79	0.019
FATTY_ACID_METABOLISM	metabolic	8	-1.70	0.023
**BDE-209**				
IL2_STAT5_SIGNALING	signaling	7	-2.05	0.0062
HEME_METABOLISM	metabolic	6	1.89	0.0063
MYC_TARGETS_V1	proliferation	23	-1.89	0.013
PI3K_AKT_MTOR_SIGNALING	signaling	9	1.84	0.012
UV_RESPONSE_UP	DNA damage	12	1.74	0.023
OXIDATIVE_PHOSPHORYLATION	metabolic	9	-1.71	0.046
COMPLEMENT	immune	7	-1.69	0.016
INFLAMMATORY_RESPONSE	immune	8	-1.63	0.035
**MIX**				
E2F_TARGETS	proliferation	32	3.38	<0.0001
G2M_CHECKPOINT	proliferation	33	3.05	<0.0001
MYC_TARGETS_V2	proliferation	10	2.30	<0.0001
MYC_TARGETS_V1	proliferation	32	2.05	0.0060
TNFA_SIGNALING_VIA_NFKB	signaling	32	-2.04	0.0058
MITOTIC_SPINDLE	proliferation	20	2.04	0.0020
BILE_ACID_METABOLISM	metabolic	12	1.93	0.0059
MTORC1_SIGNALING	signaling	39	1.90	0.014
ESTROGEN_RESPONSE_LATE	signaling	27	1.81	0.014
ANGIOGENESIS	development	5	1.71	0.034
FATTY_ACID_METABOLISM	metabolic	16	1.67	0.026
